# A Wireless Visualized Sensing System with Prosthesis Pose Reconstruction for Total Knee Arthroplasty

**DOI:** 10.3390/s19132909

**Published:** 2019-07-01

**Authors:** Hanjun Jiang, Shaolin Xiang, Yanshu Guo, Zhihua Wang

**Affiliations:** Institute of Microelectronics, Tsinghua University, Beijing 100084, China

**Keywords:** total knee arthroplasty (TKA), multimodal sensing, visualization, pose reconstruction

## Abstract

The surgery quality of the total knee arthroplasty (TKA) depends on how accurate the knee prosthesis is implanted. The knee prosthesis is composed of the femoral component, the plastic spacer and the tibia component. The instant and kinetic relative pose of the knee prosthesis is one key aspect for the surgery quality evaluation. In this work, a wireless visualized sensing system with the instant and kinetic prosthesis pose reconstruction has been proposed and implemented. The system consists of a multimodal sensing device, a wireless data receiver and a data processing workstation. The sensing device has the identical shape and size as the spacer. During the surgery, the sensing device temporarily replaces the spacer and captures the images and the contact force distribution inside the knee joint prosthesis. It is connected to the external data receiver wirelessly through a 432 MHz data link, and the data is then sent to the workstation for processing. The signal processing method to analyze the instant and kinetic prosthesis pose from the image data has been investigated. Experiments on the prototype system show that the absolute reconstruction errors of the flexion-extension rotation angle (the pitch rotation of the femoral component around the horizontal long axis of the spacer), the internal–external rotation (the yaw rotation of the femoral component around the spacer vertical axis) and the mediolateral translation displacement between the centers of the femoral component and the spacer based on the image data are less than 1.73°, 1.08° and 1.55 mm, respectively. It provides a force balance measurement with error less than ±5 N. The experiments also show that kinetic pose reconstruction can be used to detect the surgery defection that cannot be detected by the force measurement or instant pose reconstruction.

## 1. Introduction

**Figure 1:**
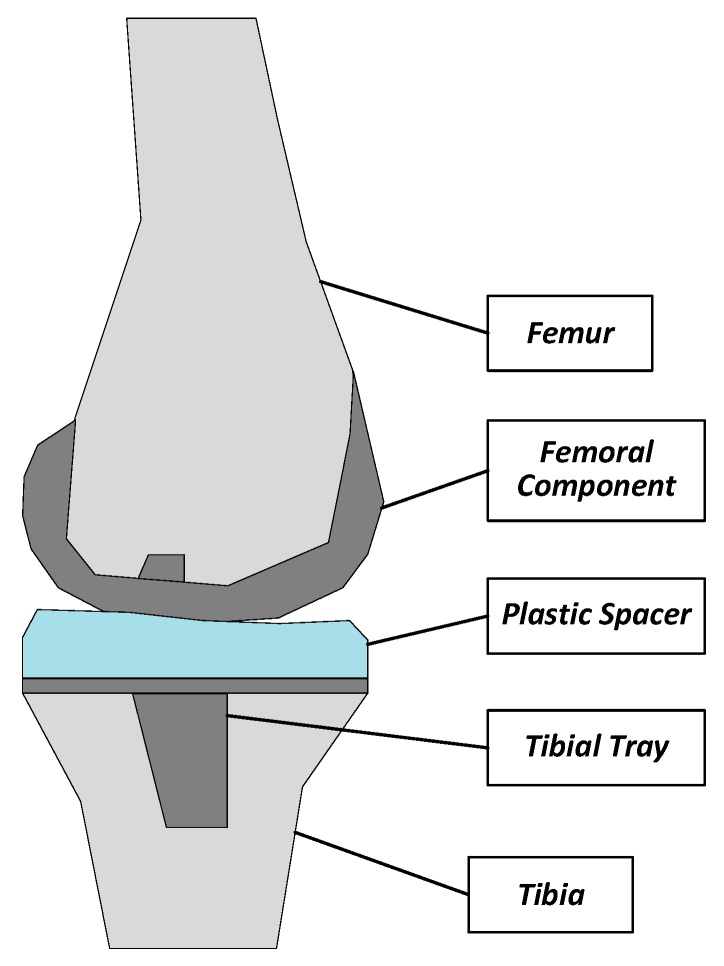
A total knee replacement prosthesis (side view).

There is an increasing number of people that suffer from arthritis or chronic joint diseases. For example, in the United States, the number of people aged 65 and older with these symptoms will exceed 41 million by 2030 [1]. Among them, knee arthritis contributes considerably to morbidity and results in a low quality of life. The primary surgical treatment for these patients is the total knee arthroplasty (TKA), namely, the replacement of the worn knee joint with a prosthesis implant [2,3,4,5,6]. As shown in Figure 1, the TKA implant is composed of three components: the femoral component, the tibia component and the polyethylene plastic spacer. 

A successful TKA surgery improves the knee function, and relieves the knee joint pain. However, the improper placement of the knee prosthesis may accelerate the attrition of the plastic spacer, which will result in a reduced working life, and cause severe pain to patients [2]. The integrity of a successful TKA surgery depends on several factors, including the appropriate alignment of the components, the rotational congruency between the prosthetic proximal tibia and the prosthetic femoral condyles, and the ligamentous balance of the knee joint [3]. Recent research [4] shows that the overall TKA failure rate is about 2.3%, and more than 74% of TKA failures are caused by non-infection reasons such as instability, aseptic loosening, stiffness, etc. Actually, most TKA failures are directly or indirectly caused by the operative mechanical reasons such as malalignment [5]. Traditionally, the quality of the TKA surgery is determined exclusively by the experience of the surgeons. The digitally guided assistance equipment will be meaningful to improve the surgery quality, and reduce the failure rate caused by the operative reasons.

Many efforts have been made to develop the instrumentation of the knee joint prosthesis, for the purpose of surgery quality evaluation or long-term monitoring. Some investigations focused on measuring the contact force inside the joint prosthesis under different conditions [7,8,9,10,11,12,13,14,15]. In [7,8,9], a tibia tray was designed to measure the six load components in TKA. The in-vivo experiment under the conditions of walking or stair climbing during the postoperative follow-up of 6 to 10 months was conducted, in which the contact force of the prosthesis components were measured for five subjects. Similarly, a set of force sensing components were measured in [10,11,12,13] for three subjects in the scenarios of exercising and recreational activities after the TKA surgery, and the in-vivo experiment validated the effectiveness of the method. An implantable tibia prosthesis with the multiaxial force sensing was implemented and reported in [13]. In [14], a sensing system inside the knee implant for the force measurement was designed, fabricated and tested. The sensing system could be used to measure the contact force up to 1.5 times the body weight, and it was suitable for long-term monitoring. In [15,16,17], an instrumented smart knee prosthesis for the in-vivo measurement of the contact force and kinematics was proposed. This system could be used to monitor the knee prosthesis after the implantation with three magnetic sensors and a permanent magnet, and to prevent the possible damage to the prosthesis by detecting the load imbalance or abnormal forces and kinematics in the knee prosthesis. In [18], a long-term knee implant fatigue monitoring system using a floating-gate sensor array was introduced for the long-term, battery-less fatigue monitoring. All the above-mentioned designs were successful in measuring knee prosthesis contact force, and some could also measure the kinetic movement of the knee prosthesis. Nevertheless, they were designed to monitor the knee prosthesis after the TKA surgeries. It is also very meaningful to develop the technique to improve the implantation surgery quality during the process of TKA surgeries. The motivation of this work is to provide a device to help the surgeons to improve the TKA surgery quality during the surgery process. Such a device is used only during the TKA surgery for measurement of less than one hour. 

Some efforts have been made to provide the auxiliary methods to help the surgeons to improve the quality in the surgery procedure. In [19], a “VERASENSE Knee System” is presented, in which an array of sensors provides the dynamic, intraoperative feedback regarding tibiofemoral position and quantitative pressure at peak contact points in the medial and lateral compartments in the TKA surgery. The kinematic tracking can also be assessed. In [20], a wireless knee joint force measurement system is proposed to increase the accuracy of the ligament balancing procedure. In [21], a force amplitude- and location-sensing device has been designed to improve the ligament balancing procedure in TKA. However, the above-mentioned systems only acquire the single modal sensor data, which may miss some important information. The sensing system can be more comprehensive with additional sensor data such as the image data. 

A wireless visualized sensing system has been proposed and implemented in this work for multimodal signal sensing inside the knee joint prosthesis. The system is used during the surgery as a trial component for adjustment and calibration of the implant without reforming the standard knee prosthesis or changing the standard clinical procedures. The proposed system consists of a small-sized sensing device, a wireless data receiver and a data processing workstation. The system can be used to acquire both the direct images and the contact force distribution inside the prosthesis. The real-time images can help the surgeons directly see and understand the real situation in the joint. More importantly, the image sensors have higher resolution than the piezoelectric, magneto-resistive and other types of physical sensors, and the proposed system can acquire much richer information about the knee joint prosthesis with the vision sensing.

The proposed sensing device is used during the surgery when the patient lies flat. Once the sensing device is placed in the knee joint, the surgeons will move the shin manually, and the device records the inside images and the contact forces. The further signal processing reconstructs the instant pose, and the kinematic trajectory of the relative movement between the femoral component and spacer. The surgeons can use the obtained information to calibrate the position of the components in the joint prosthesis. During the surgery, the sensing device is temporarily inserted into the knee implant as a trial component and will be replaced by the real spacer component once the femoral component and tibia component are fitted to the proper position. The influence on the TKA procedure is quite small. The TKA surgeons can easily use it in the standard clinical procedures. In general, the proposed sensing device cannot be used to monitor the progression of the prosthesis over time after the surgery, partially due to the limited battery lifetime with the huge power consumption associated with image data acquisition and processing.

The rest of the paper is organized as follows. In Section 2, the proposed system architecture and the design considerations are presented. Section 3 describes the hardware design details of the sensing device as well as the wireless data receiver. In Section 4, the detailed data processing procedures and algorithms will be introduced. Section 5 gives the experimental results. This work is concluded in Section 6.

## 2. Design Considerations and System Design

**Figure 2:**
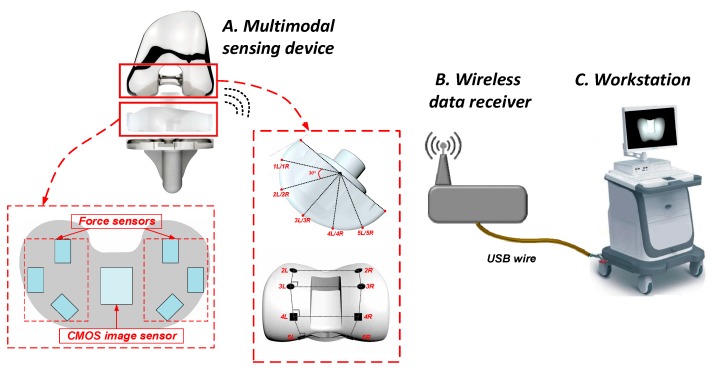
Proposed system architecture.

The proposed wireless sensing measurement system consists of three parts, namely, the multimodal sensing device, the wireless data receiver, and the workstation, as shown in Figure 2.

The small-sized multimodal signal sensing device has the identical shape and size as the real spacer of the knee joint prosthesis. During the surgery, before the final placement of the real spacer, the sensing device is placed in its position inside the joint prosthesis as a trial of the spacer. The sensing device can take two types of data: (1) the images of the femoral component viewed from the spacer side; (2) the contact force distribution between the spacer and the femoral component. To facilitate the image data processing, some simple but easily recognizable markers are printed on the surface of the femoral component.

The wireless data receiver is used to receive the sensing data acquired by the sensing device through a 432 MHz wireless link. The data receiver is then connected with the workstation through a USB cable. The signal processing will be carried out in the workstation, which will recognize the real-time relative position between the spacer and the femoral component from the multimodal sensing data. The algorithm implemented in the workstation can also the 3D kinematic trajectory of the femoral component with respect to the spacer. The surgeons can use the reconstructed information to evaluate if the knee joint prosthesis is properly installed, and make the adjustment if necessary.

Generally speaking, the force data acquired through this system is only 2D data, and it is difficult to obtain the 3D information from the force data only. The image data is also 2D data, but the high-resolution images also contain the depth information. With the proposed kinetic pose estimation algorithm in this work, the presented system can output a 3D motion trajectory which is not available from other systems that acquire the force data only. 

**Figure 3:**
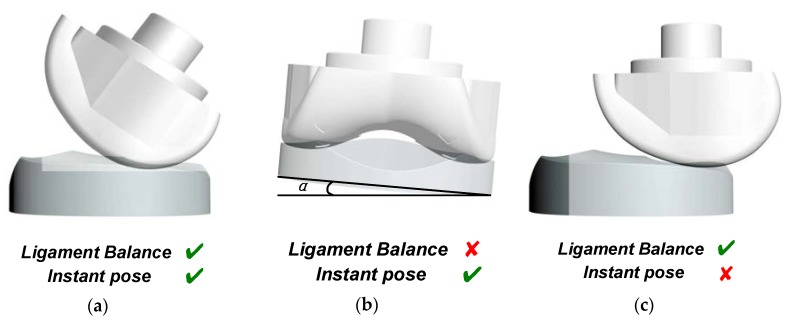
Typical situations of the knee joint prosthesis of total knee arthroplasty (TKA) surgery: (**a**) the joint prosthesis is well installed, (**b**) inappropriate condition with the spacer tilted, (**c**) inappropriate condition with malalignment between the spacer and the femoral component.

There are some abnormal TKA situations that cannot be identified using the force data only, and there are also some abnormal situations that cannot be detected only using the image data. Figure 3 gives some examples to show the advantage of multimodal sensing data instead of the single-modal data. As shown in Figure 3a, if the knee joint is appropriately installed, the spacer and the femoral component is well aligned with a certain relative pose in both the static and kinetic conditions, and the ligament around the two sides of the knee joint is well balanced. The sensing device presented in [21] could be used to determine if the ligament is well balanced by comparing the contact forces of the two sides, since it could only measure the contact force distribution between the spacer and the femoral component. Such a device could detect the inappropriate condition as shown in Figure 2b, in which the spacer is tilted, and the contact force distribution is not in equilibrium if the patients stand straight. However, the sensing device in [21] could not report the inappropriate situation as shown in Figure 2c. In this situation, the contact forces between the two sides of the knee joint are equal, and therefore the device in [21] would faultily judge it as a good situation, although the spacer and the femoral component are misaligned. Nevertheless, the malalignment can be detected by processing the direct images taken inside the joint using the multimodal sensing device presented in this work. In general, the surgery flaws shown in both Figure 3b,c can be detected using the presented multimodal sensing system.

## 3. Hardware Implementation

The hardware implementation of the presented sensing system will be presented in this section.

### 3.1. Multimodal Sensing Device

**Figure 4:**
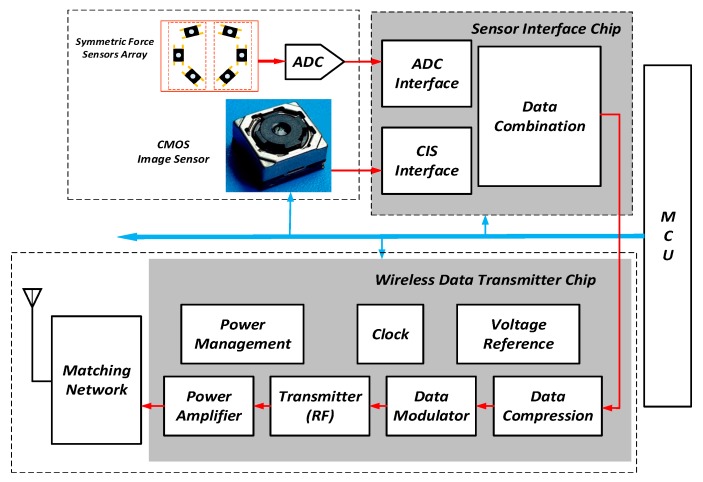
Functional diagram of the multimodal sensing device.

The functional diagram of the image and force sensing device is shown in Figure 4. It consists of five key functional blocks, i.e., the image sensor, the force sensors array in together with an analog-to-digital converter (ADC) for signal conversion, the wireless data transmitter, the sensor interface chip to bridge the sensors and the transmitter, and the microcontroller (MCU) for the system initialization. There are four white LEDs distributed evenly around the image sensor lens. These four LEDs provide the necessary lighting for the image sensing, since the sensing device will work in the dark or dim environment side the knee joint. The illuminance can be tuned by adjusting the driving current of the LEDs to avoid the overexposure or underexposure. 

The MSP430 MCU by Texas Instruments Inc. provides the initial settings for all the other chips in the sensing device during the power-up phase. Since the wireless data transmitter and the sensors operate autonomously, the MCU is programmed to power off to save power consumption once the device starts the full operation.

The OV7670 image sensor used in this device is a CMOS sensor manufactured by Omnivision with a maximum resolution of 640 × 480. In this application, it can be programmed to output images with 480 × 480 or 240 × 240 resolution. A view angle of 140° is achieved by using a specially designed wide-angle macro lens. Since the image sensor is very close to the femoral component, this wide view angle macro lens can guarantee that most of the surface of the femoral component can be “seen” by the image sensor.

The sensing device contains six low profile force sensors manufactured by Honeywell based on silicon-implanted piezo resistors. Each sensor has a measurement range of 0–45 N. A 6-channel 24-bit ΣΔ ADC by ADI is used to quantize the force sensors’ output. 

The wireless data transmitter chip is a highly integrated system-on-a-chip (SoC) which is an improved design of that reported in [22]. The ultra-low power SoC was originally designed to transmit the image sensor data. It is mainly composed of a minimum shift keying (MSK) transmitter working at the 400 MHz band and an image data compressor. In this application, the transmitter is configured to work at 416 MHz, and it provides a raw data rate of 3 Mbps. The image data compressor provides a near-lossless data compression ratio of ~3, which can help to boost the transmission frame rate. In this application, the transmitter SoC can be configured to transmit 480 × 480 images with a frame rate of ~3 fps, or 240 × 240 images with a frame rate of 6–8 fps. The SoC contains one charge-pump boost regulator and three programmable low-dropout (LDO) linear regulators, which provide 4 V, 2.5 V, 1.8 V and 1.2 V power supplies to all the other circuits in the SoC and the other function blocks in the sensing device. The 4V power supply is used to drive four white LEDs, which serve as the flash lights for image sensing. A coil antenna with −8.9 dBi peak gain and a voltage standing wave ratio (VSWR) of less than 2.0 at the 416 MHz center frequency with the proper matching between the antenna and the RF power amplifier. The RF transmitter has an energy efficiency of 1.3 nJ/bit not including the power amplifier.

Since the wireless transmitter SoC was originally designed to transmit the image data, it can only receive the data from the image sensor using an 8-bit parallel data port. The dedicated sensor interface chip is used to pack the sensing data from the force sensor array and the image sensor into the format of 8-bit parallel image data, which is readable by the SoC. Actually, the first four data rows of each 480 × 480 or 240 × 240 image is replaced by the force sensor data with careful consideration of data synchronization. From the view point of the transmitter SoC, it just receives and transmits the “image” data from the interface chip. By doing so, each frame of the image will lose the first four rows, which is affordable, considering that each image contains at least 240 rows.

The sensing device is supplied by a 3 V lithium manganese battery with a capacity of 170 mAh. There is the potential risk to use this type of battery since it is not dedicated for the medical applications. Regardless, this battery is only used for the experiments before the clinical trial. In the future, the lithium manganese battery can be replaced by a safer battery, such as the lithium/iodine cell battery for the real medical product. The currently used battery has a peak current of 30 mA that occurs when the image sensor is enabled, with the image sensor drawing 15 mA from a regulated 2.5 V supply and the flash lights consuming ~14 mA current. The transmitter SoC has a peak current of ~6 mA. Note that the image sensor and the transmitter are not enabled simultaneously to avoid too much peak current. The force sensors and the ADC has a peak current of less than 1 mA. In overall, the sensing device consumes a peak current of ~30 mA.

All the circuits in the sensing device are powered on only when necessary, the sensing device has an average current of ~10 mA from the 3 V supply. Roughly, 40% of the total average current is contributed by the transmitter SoC and almost all the remaining 60% is contributed by the image sensor in together with its flash lights. The force sensors and the following ADC are enabled at very low duty cycle ratio, and the contribution to the average current is less than 1%.

**Figure 5:**
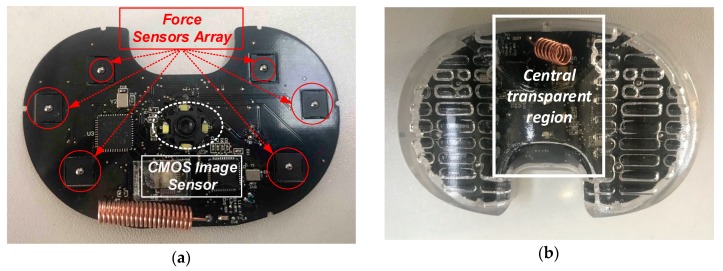
Package and assembling of the sensing device. (**a**) printed circuit board (PCB), (**b**) the entire sensing device with the transparent shell.

As shown in Figure 5a, the force sensors and the image sensor reside on the same printed circuit board (PCB) which has the same outline as the spacer. The PCB is sealed inside a transparent shell which is composed of the upper shell and lower shell. The central region of the upper shell is transparent and polished to ensure the image quality. The entire sensing device is shown in Figure 5b. It has the identical shape and size as the real spacer used in the knee joint prosthesis. Since there are many versions of spacers with various sizes to meet the requirements of different patients, the sensing devices in the real product should also have as many as versions to match the spacers. The shell of the sensing device is made of the medical-level polycarbonate. Since the device is not permanently implanted, the wear issue is not of concern. Note that the sensing device is used when the patient lies flat. It is estimated that the force applied to the shell of the device is less than 20 kilograms. The mechanical architecture of the sensing device is well designed so that it can tolerate the loads up to 50 kilograms. Experiments have been performed to validate the mechanical reliability of the sensing device with a 50 kg load.

The performance of the sensing device is summarized in Table 1.

### 3.2. Wireless Data Receiver

**Figure 6:**
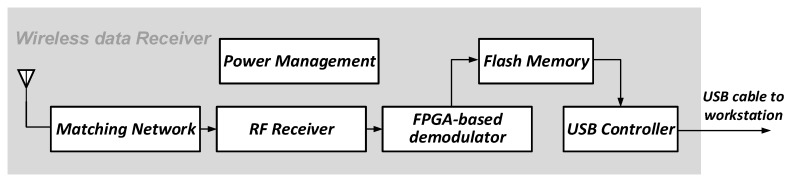
Block diagram of the data receiver.

The data receiver in the proposed wireless visualized measurement system is used to receive the multimodal data from the sensing device. The block diagram of the receiver is shown in Figure 6. The key parts of the data receiver are the RF receiver and the FPGA-based MSK demodulator. The digital demodulator receives the digitized intermediate frequency (IF) signals from a pair of 8-bit 24 Msps ADCs, and performs the MSK demodulation. Note that in the sensing device, the image sensor output and the force sensors’ output are packed. Correspondingly, the image data and the force data are decomposed from the received data in the data receiver. The data receiver is then connected to the workstation through a USB cable for further data processing.

**Figure 7:**
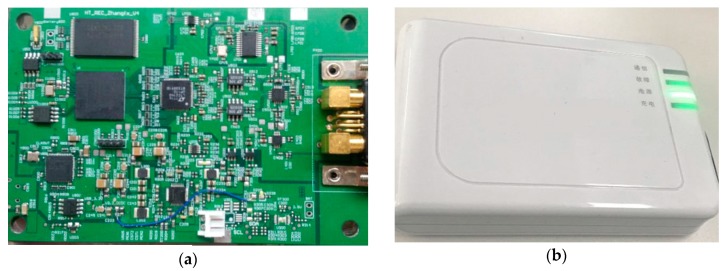
PCB and package of the data receiver.

The PCB and package of the data receiver is shown in Figure 7. The data receiver has a size of 124 × 86 × 22 mm^3^. It contains a li-iron battery of 4000 mAh, and the battery life is about 10 h.

## 4. Multimodal Sensor Data Processing

The multimodal sensing data is eventually sent to the workstation for further processing to acquire the relative pose of the components in the knee joint prosthesis.

**Figure 8:**
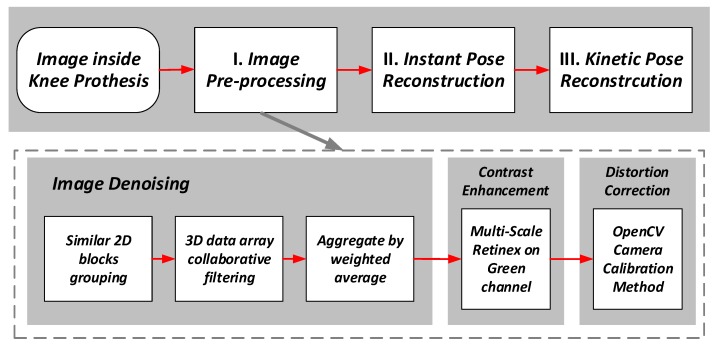
Data processing flow in the workstation.

The force sensors’ data can be used to check the ligament balance of the knee prosthesis, and the details of the force data processing can be found in the paper previously published by our group [21]. As shown in Figure 8, the image data processing is composed of three steps, i.e., the image data pre-processing, the instant pose reconstruction from the individual image, and the kinetic pose reconstruction based on multiple consecutive images.

### 4.1. Image Pre-Processing

In the image pre-processing part, the images are denoised, and then the contrast is enhanced, and the lens distortion is corrected. Note that the contrast enhancement is necessary since all the images are taken under the low illumination scenario inside the knee prosthesis. The distortion introduced by the wide-angle macro-lens must also be corrected before further processing. The steps of the pre-processing are shown in Figure 8.

The Block-Matching and 3D filtering (BM3D) algorithm [23] is used for the denoising. In this algorithm, the similar blocks in the femoral component surface images are used to eliminate the noise, and the image quality is improved in terms of both the peak signal-to-noise ratio (PSNR) and the subjective visual quality.

For the contrast enhancement, a modified Multi-Scale Retinex (MSR) algorithm [24] is used to enhance the edge features of the images. For each input image data matrix *S*(*x*,*y*), the output of the MSR processing is the reflectance matrix *R*(*x*,*y*) given by:

(1) R(x,y)=S(x,y)/(F(x,y)∗S(x,y))

in which *F*(*x*,*y*) is a Gaussian filter. *F*(*x*,*y*) is given by:

(2) $$F(x,y)=\exp\left(-\left(x^{2}+y^{2}\right)/c^{2}\right)$$

where c is the scale factor. To simplify the calculation, the logarithmic matrix *r*(*x*,*y*) is used, and *r*(*x*,*y*) is calculated as:

(3) r(x,y)=lnR(x,y)=lnS(x,y)−ln(F(x,y)∗S(x,y))

Following the method in [25], three Gaussian filters $F_{k}\left(x,y\right)=\exp\left(-\left(x^{2}+y^{2}\right)/c_{k}^{2}\right),\;k=1,2,3$ are used to generate three outputs, i.e., $r_{k}\left(x,y\right),\;k=1,2,3$ and the weighted summation of $r_{k}\left(x,y\right),\;k=1,2,3$ is calculated to find the final MSR output.

(4) $$R\left(x,y\right)=\exp\left(\sum_{k=1}^{3}W_{k}r_{k}\left(x,y\right)\right)$$

In this application, based on the experiments on the image data taken in the emulated environment, the MSR processing is only applied to the G channel of the original RGB image data, which yields the best subjective quality. Also based on the experimental data, the following set of parameters are used for the MSR processing, i.e., *c*_1_ = 15, *c*_2_ = 80, *c*_3_ = 250, and *W*_1_ = *W*_2_ = *W*_3_ = 1/3.

The R, G and B channels after the MSR processing are normalized separately, to balance the relative intensities of the three-color channels.

The last step in the pre-processing is to correct the lens distortion. The camera calibration method from OpenCV [26] is used. A test board with the chess board pattern is utilized to find the camera matrix, distortion coefficients, rotation and translation vectors, etc., which is then used for the distortion correction.

### 4.2. Establishment of the Pose Reconstruction Problem

**Figure 9:**
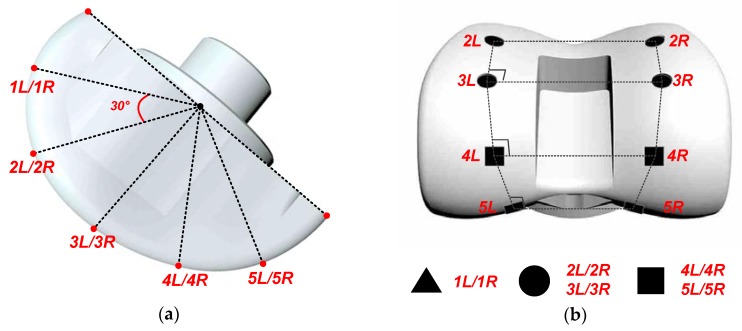
Femoral component with five pairs of control points on its surface: (**a**) side view, (**b**) front view (partial).

To facilitate the image data processing, five pairs of control points are marked on the femoral component, by printing some special markers on its surface. Those control points are numbered as *1L/1R, 2L/2R, 3L/3R, 4L/4R* and *5L/5R*. As shown in the side view of the femoral component given in Figure 9, the five pairs of control points are spaced approximately evenly on the femoral component surface with an angle difference of 30°. Any two pairs of control points form a rectangle. The femoral component is usually made of reflective materials, which will bring difficult to take the. However, the most concerned features of the images are the five pairs of control points. Non-reflective material with high contrast should be chosen to print the control points, so that they can then easily be distinguished, even if the entire image is affected by the surface reflection issue. 

Only three types of markers are used, namely, the triangle, round and square markers. 1*L*/1*R* markers are triangle, 2*L*/2*R* and 3*L*/3*R* markers are round, and 4*L*/4*R* and 5*L*/5*R* markers are square. Since the movement of the femoral component has limited freedom, the control point numbers can be easily recognized by recognizing the shapes of the markers. For example, if the surface image of the femoral component contains two round markers and two square markers, the control point numbers are recognized as 3*L*/3*R* and 4*L*/4*R*.

The image data processing of this system turns to finding the relative position between the spacer (sensing device) and the femoral component from the surface images of the femoral component “seen” by the spacer (sensing device) with the aid of the control points. 

**Figure 10:**
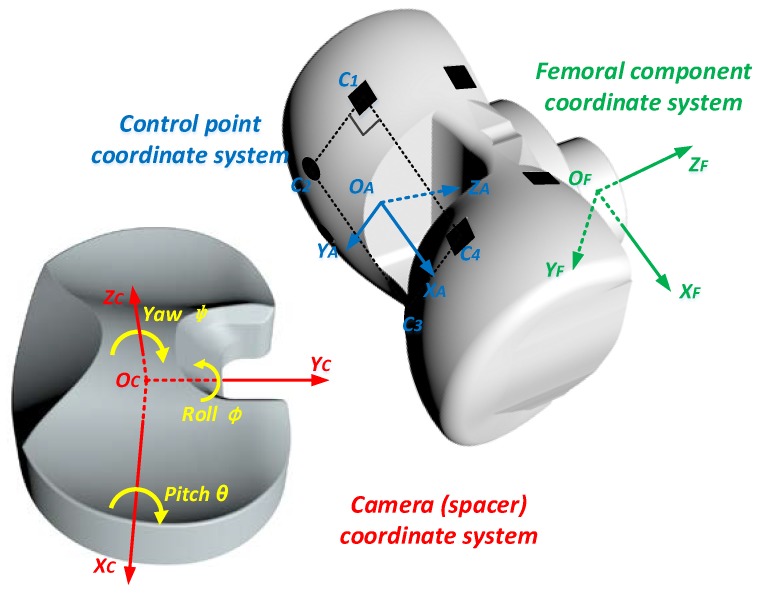
The coordinate systems to represent the relative position between the spacer and the femoral component.

Geometrically, the spacer and the femoral component are represented by two coordinate systems, namely, the spacer coordinate system {*O*_C_*X*_C_*Y*_C_*Z*_C_}, and the femoral coordinate system {*O*_F_*X*_F_*Y*_F_*Z*_F_}, as shown in Figure 10. For the spacer coordinate system, the origin *O*_C_ is the center of the space bottom plane, the X axis *O*_C_*X*_C_ is defined as the long axis the space bottom plane, and the Y axis *O*_C_*Y*_C_ is the axis perpendicular to *O*_C_*X*_C_ on the space bottom plane, and the Z axis *O*_C_*Z*_C_ is vertically perpendicular to the space bottom plane. Ideally, when the femur is alignment with the tibia, the femoral coordinate system {*O*_F_*X*_F_*Y*_F_*Z*_F_} is fully parallel to {*O*_C_*X*_C_*Y*_C_*Z*_C_}, and the two origins *O*_F_ and *O*_C_ are apart vertically. The spacer coordinate system is used as the camera coordinate system in this implementation. The relative position of the spacer and the femoral component can be described by the rotational angles [*ϕ*, *θ*, *ψ*], and the 3-dimensional translation *t*_CF_ = [*t*_x_, *ty*, *t*_z_]^T^ between the two origins. *ϕ* is the the roll angle about the *O*_C_*Y*_C_ axis. *θ* is the flexion-extension rotation angle, namely, the pitch angle about the *O*_C_*X*_C_ axis. *ψ* is the internal–external rotation angle, namely, the yaw angle about the *O*_C_*Z*_C_ axis between the two coordinate systems. *t*_x_ is the mediolateral translation, namely, the horizontal distance between the centers of the femoral component and the spacer along the *O*_C_*X*_C_ axis. For a successful TKR surgery, it is expected that *ϕ*, *ψ* and *t*_x_ are all zero when the tibia moves relative to the femur.

In the image data processing, it is more convenient to use the rotation matrix R instead of the rotational angles [*ϕ*, *θ*, *ψ*]. [*ϕ*, *θ*, *ψ*] can easily calculated from *R* based on the definition of *R* as follows:

(5) $$R_{CF}=\left[\begin{array}{ccc} \cos\phi \cos\psi & -\cos\phi \sin\psi & \sin\phi \\ \cos\theta \sin\psi +\cos\psi \sin\phi \sin\theta & \cos\psi \cos\theta -\sin\phi \sin\psi \sin\theta & -\cos\phi \sin\theta \\ -\cos\psi \cos\theta \sin\phi +\sin\psi \sin\theta & \cos\theta \sin\phi \sin\psi +\cos\psi \sin\theta & \cos\phi \cos\theta \end{array}\right]$$

The relative pose reconstruction problem is converted to the problem to find R_CF_ and t_CF_ between two coordinate systems {*O*_C_*X*_C_*Y*_C_*Z*_C_} and {*O*_F_*X*_F_*Y*_F_*Z*_F_}.

It is not easy for the sensing device to directly recognize the coordinate system {*O*_F_*X*_F_*Y*_F_*Z*_F_}. However, with the wide view angle of the image sensor in the sensing device, any femoral component image taken by the sensing device contains at least 2 pairs of control points, which can define an assistant control point coordinate system {*O*_A_*X*_A_*Y*_A_*Z*_A_}. There are a series of control point coordinate systems {*O*_A_*X*_A_*Y*_A_*Z*_A_} defined by the control points, and the rotation matrix R_AF_ and the translational distance t_AF_ between any {*O*_A_*X*_A_*Y*_A_*Z*_A_} and {*O*_F_*X*_F_*Y*_F_*Z*_F_} are exactly known. On the other hand, R_CA_ and t_CA_ between {*O*_C_*X*_C_*Y*_C_*Z*_C_} and {*O*_A_*X*_A_*Y*_A_*Z*_A_} can be calculated using the image data processing shown next. 

Assume that the coordinates of a given point in these three coordinate systems are *C*_C_ = [*x*_C_, *y*_C_, *z*_C_]^T^, *C*_A_ = [*x*_A_, *y*_A_, *z*_A_]^T^, and *C*_F_ = [*x*_F_, *y*_F_, *z*_F_]^T^, respectively. The coordinate transformation gives the following equations:

(6) $$C_{F}=R_{CF}\left(C_{C}-t_{CF}\right)$$

(7) $$C_{F}=R_{AF}\left(C_{A}-t_{AF}\right)$$

(8) $$C_{A}=R_{CA}\left(C_{C}-t_{CA}\right)$$

Substituting (6) and (7) into (8) gives:

(9) $$C_{F}=R_{AF}\left(R_{CA}\left(C_{C}-t_{CA}\right)-t_{AF}\right)\Rightarrow C_{F}=R_{AF}R_{CA}\left(C_{C}-\left(t_{CA}+R_{CA}^{-1}t_{AF}\right)\right)$$

Comparing (6) and (9), it follows that:

(10) $$\left[\begin{array}{cc} R_{CF} & t_{CF} \end{array}\right]=\left[\begin{array}{cc} R_{AF}R_{CA} & R_{CA}^{-1}t_{AF}+t_{CA} \end{array}\right]$$

The problem to solve *R*_CF_ and *t*_CF_ is finally converted to the coplanar perspective-4-points problem [27] to find [*R*_CA_, *t*_CA_], with [*R*_AF_, *t*_AF_] as the known parameters.

### 4.3. Instant Pose Reconstruction

After the pre-processing, the control points in the femoral component images are recognized. The images are firstly filtered by an adaptive threshold filter to generate the binary images. The contours of the control point markers are then recognized using the method by [28]. The numbers of the control points are recognized by the markers’ shapes. For each individual image, four adjacent control points which form the biggest rectangle area in this image are chosen to establish the control point coordinate system {*O*_A_*X*_A_*Y*_A_*Z*_A_}.

An analytic and non-iterative method is then proposed to solve the coplanar perspective-4-points problem to find [*R*_CA_, *t*_CA_] as described in the previous subsection. There are many classical methods to solve this perspective problem, including the non-iterative method Epnp [29], the iterative linear solution using unbiased statistics [30,31], some simple methods based on P3P problem [32,33] and many linear analytic solutions [34,35,36,37,38] and so on. Compared to these classical methods, the proposed method has the advantage of low computational complexity.

**Figure 11:**
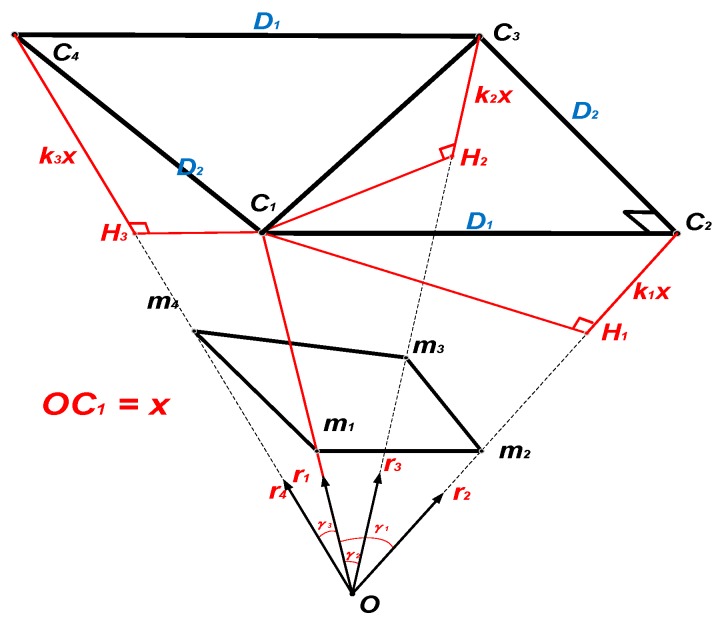
Geometry of the proposed analytic and non-iterative method.

The geometry of the proposed method is explained in Figure 11. As shown in Figure 11, the four control points *C*_1_, *C*_2_, *C*_3_, *C*_4_ are projected to the image plane as the image points *m*_1_, *m*_2_, *m*_3_, *m*_4_, and the correspondence between the control points and the image points is known. The point *O* is the perspective center of the camera. Solving the estimation problem can be equivalent to solving the depth of *OC*_1_, *OC*_2_, *OC*_3_, and *OC*_4_. The vector $\vec{r_{i}}$ is the unit vector with the same direction as $\vec{Om_{i}}$ (I = 1, 2, 3, 4) and $\gamma_{1}$, $\gamma_{2}$, $\gamma_{3}$ are the angles between $\vec{r_{1}}$ and $\vec{r_{2}}$, $\vec{r_{3}}$, $\vec{r_{4}}$, respectively. H_1_, H_2_, H_3_ are the points on line *OC*_2_, *OC*_3_, and *OC*_4_ with $C_{1}H_{1}⊥OC_{2}$, $C_{1}H_{2}⊥OC_{3}$ and $C_{1}H_{3}⊥OC_{4}$.

To simplify this problem, assume that *OC*_1_ has a length of *x* and *C*_1_*H*_1_, *C*_1_*H*_2_, *C*_1_*H*_3_ equal to *k*_1_*x*, *k*_2_*x* and *k*_3_*x* respectively. How to find the values of *k*_1_, *k*_2_, *k*_3_ and *x* is explained as follows. In the triangle *C*_1_*C*_2_*C*_3_:

(11) $$C_{1}C_{2}{}^{2}=D_{1}^{2}=C_{1}H_{1}{}^{2}+H_{1}C_{2}{}^{2}=x^{2}\left(\sin^{2}\gamma_{1}+k_{1}^{2}\right)$$

(12) $$C_{1}C_{3}{}^{2}=D_{1}^{2}+D_{2}^{2}=C_{1}H_{2}{}^{2}+H_{2}C_{3}{}^{2}=x^{2}\left(\sin^{2}\gamma_{2}+k_{2}^{2}\right)$$

where *D*_1_ and *D*_2_ are the two side lengths of rectangle *C*_1_*C*_2_*C*_3_*C*_4_. According to the cosine theorem:

(13) $$\cos∠C_{2}C_{1}C_{3}=\frac{D_{1}}{\sqrt{D_{1}^{2}+D_{2}^{2}}}=\frac{\vec{C_{1}C_{2}}*\vec{C_{1}C_{3}}}{\left|\vec{C_{1}C_{2}}\right|*\left|\vec{C_{1}C_{3}}\right|}=\frac{\vec{C_{1}C_{2}}*\vec{C_{1}C_{3}}}{D_{1}/\sqrt{D_{1}^{2}+D_{2}^{2}}*C_{1}C_{3}{}^{2}}$$

According to the geometry relationship:

(14) $$\vec{C_{1}C_{2}}=\vec{OC_{2}}-\vec{OC_{1}}=x\left[\left(\cos\gamma_{1}+k_{1}\right)\vec{r_{2}}-\vec{r_{1}}\right]$$

(15) $$\vec{C_{1}C_{3}}=\vec{OC_{3}}-\vec{OC_{1}}=x\left[\left(\cos\gamma_{2}+k_{2}\right)\vec{r_{3}}-\vec{r_{1}}\right]$$

Substitute (14) and (15) into (13):

(16) $$\frac{D_{1}^{2}}{D_{1}^{2}+D_{2}^{2}}\left(\sin^{2}\gamma_{2}+k_{2}^{2}\right)x^{2}=x^{2}\left[\left(\cos\gamma_{1}+k_{1}\right)\vec{r_{2}}-\vec{r_{1}}\right]\left[\left(\cos\gamma_{2}+k_{2}\right)\vec{r_{3}}-\vec{r_{1}}\right]$$

(16) can be expanded to (17):

(17) $$a_{1}k_{2}^{2}+b_{1}k_{2}+c_{1}=k_{1}\left(d_{1}k_{2}+e_{1}\right)$$

Substitute (17) into (11)/(12):

(18) $$f_{1}k_{2}^{4}+g_{1}k_{2}^{3}+h_{1}k_{2}^{2}+i_{1}k_{2}+j_{1}=0$$

Similarly, in the triangle *C*_1_*C*_4_*C*_3_:

(19) $$a_{2}k_{2}^{2}+b_{2}k_{2}+c_{2}=k_{3}\left(d_{2}k_{2}+e_{2}\right)$$

(20) $$f_{2}k_{2}^{4}+g_{2}k_{2}^{3}+h_{2}k_{2}^{2}+i_{2}k_{2}+j_{2}=0$$

While in the triangle *C*_1_*C*_2_*C*_4_:

(21) $$C_{1}C_{2}⊥C_{1}C_{4}\Rightarrow x^{2}\left[\left(\cos\gamma_{1}+k_{1}\right)\vec{r_{2}}-\vec{r_{1}}\right]\left[\left(\cos\gamma_{3}+k_{3}\right)\vec{r_{4}}-\vec{r_{1}}\right]=0$$

Combining (17), (19) and (21) leads to an equation without *k*_1_ or *k*_3_:

(22) $$f_{3}k_{2}^{4}+g_{3}k_{2}^{3}+h_{3}k_{2}^{2}+i_{3}k_{2}+j_{3}=0$$

In (17)–(22), the coefficients *a*_i_, *b*_i_, *c*_i_, *d*_i_, *e*_i_, *f*_i_, *g*_i_, *h*_i_, *i*_i_, *j*_i_ (I = 1, 2, 3) are used without explanation. Actually, these coefficients all have the analytical expressions in terms of $\gamma_{1}$, $\gamma_{2}$, $\gamma_{3}$, which are shown in Appendix A. (18), (20) and (22) can be combined and written in the form of matrix operation:

(23) $$\left(\begin{array}{ccccc} f_{1} & g_{1} & h_{1} & i_{1} & j_{1}\\ f_{2} & g_{2} & h_{2} & i_{2} & j_{2}\\ f_{3} & g_{3} & h_{3} & i_{3} & j_{3} \end{array}\right){\left(\begin{array}{ccccc} k_{2}^{4} & k_{2}^{3} & k_{2}^{2} & k_{2} & 1 \end{array}\right)}^{T}=\left(\begin{array}{c} 0\\ 0\\ 0 \end{array}\right)$$

The unique solution of *k*_2_ can be found from these homogeneous linear equations, based on the right singular vectors of null space of the 3 × 5 matrix [20]. Consequently, *k*_1_, *k*_3_ and length *x* can be calculated from (17), (19) and (11), respectively. Note that:

(24) $$\{\begin{array}{cc} \vec{OC_{1}}=x\vec{r_{1}} & \vec{OC_{2}}=x\left(\cos\gamma_{1}+k_{1}\right)\vec{r_{2}}\\ \vec{OC_{3}}=x\left(\cos\gamma_{2}+k_{2}\right)\vec{r_{3}} & \vec{OC_{4}}=x\left(\cos\gamma_{3}+k_{3}\right)\vec{r_{4}} \end{array}$$

Consequently, *R*_CA_ and *t*_CA_ can be calculated as:

(25) $$\vec{t_{CA}}={\left[\begin{array}{ccc} t_{x} & t_{y} & t_{z} \end{array}\right]}^{T}=\frac{1}{4}\left(\vec{OC_{1}}+\vec{OC_{2}}+\vec{OC_{3}}+\vec{OC_{4}}\right)$$

(26) $$R_{CA}=\left[\begin{array}{ccc} \frac{\vec{C_{1}C_{2}}}{\left\|\vec{C_{1}C_{2}}\right\|} & \frac{\vec{C_{1}C_{4}}}{\left\|\vec{C_{1}C_{4}}\right\|} & \frac{\vec{C_{1}C_{2}}\times \vec{C_{1}C_{4}}}{\left\|\vec{C_{1}C_{2}}\times \vec{C_{1}C_{4}}\right\|} \end{array}\right]$$

*R*_CF_ and *t*_CF_ can then be calculated using (10), and the rotational angles [ϕ, θ, ψ] can be calculated from *R*_CF_ using (5).

Obviously, the proposed method can solve the problem without any iteration, and the computation complexity can be characterized as O(1). As a result, the instant pose reconstruction can be implemented in real time, while requiring limited computation overhead.

### 4.4. Kinetic Pose Reconstruction

**Figure 12:**
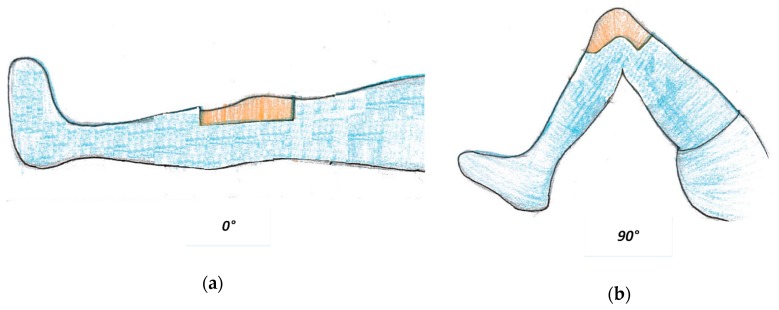
Typical angles for instant prosthesis pose check: (**a**) 0°, (**b**) 90°.

During the TKA surgeries, the instant relative pose reconstructed by the image data and the ligament balance indicated by the force sensor data should be checked by slowly moving the tibia with respect to the femur. As shown in Figure 12, there are some typical angles between the tibia and the femur, such as 0°, 45°, 90° and 130° [39].

It is also of great significance to check the kinematic trajectory of the femoral component “seen” by the spacer when moving the tibia with respect to the femur. A successful surgery will lead to a trajectory that is central symmetric and smooth. The symmetry of the kinetic trajectory indicates the balance of the prosthesis, and the smoothness indicates the knee joint can move freely without interference between the prosthesis components.

**Figure 13:**
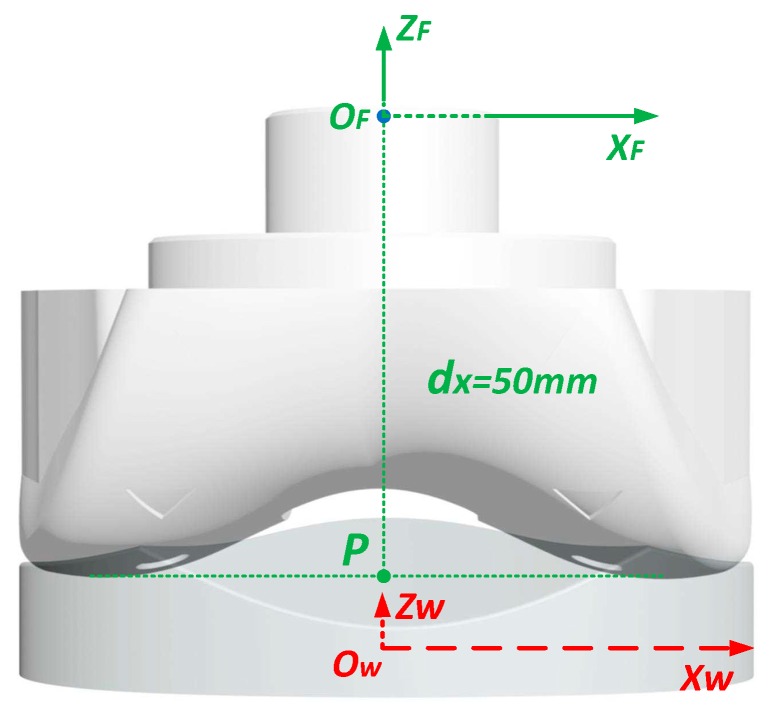
Kinetic pose reconstruction actually reconstructs the trajectory of point P in the femoral component.

In the instant pose reconstruction, the coordinate of the femoral component coordinate origin in the spacer coordinate system is described as *t*_CF_ = [*t*_x_, *t*_y_, *t*_z_]^T^. The change of *t*_CF_ = [*t*_x_, *t*_y_, *t*_z_]^T^ when moving the tibia with respect to the femur can be used as the kinetic trajectory. However, this trajectory has limited amplitude. To amplify this trajectory, another point P which is the lowest central point of the femoral component is defined and the relative trajectory of P in the spacer coordinate system is described. As shown in Figure 13, the point P has a coordinate of [0, 0, −50]^T^, then in the spacer coordinate system, the coordinate of point P is given by:

(27) $$C_{P,C}=R_{CF}^{-1}C_{P,F}+t_{CF}=R_{CF}^{-1}{\left[\begin{array}{ccc} 0 & 0 & -50 \end{array}\right]}^{T}+{\left[\begin{array}{ccc} t_{x} & t_{y} & t_{z} \end{array}\right]}^{T}$$

The kinetic pose reconstruction is just to sketch the trajectory of point P with the coordinate *C*_P,C_ in the camera coordinate system.

## 5. Experimental Results

### 5.1. Prototype Sensing System

**Figure 14:**
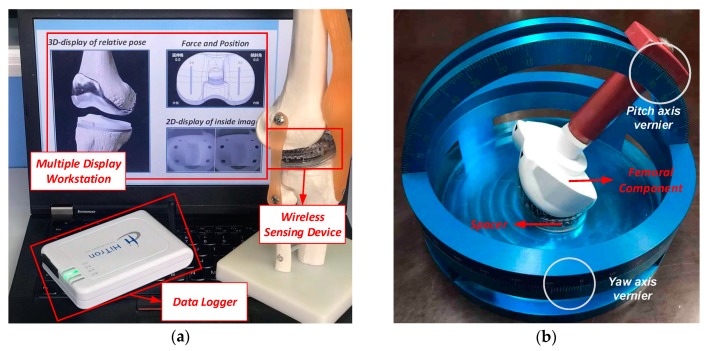
Prototype system and the test platform.

The prototype system implemented in this work is shown in Figure 14a. The sensing device can be programmed take images with resolution of 240 × 240 or 480 × 480. The frame rate of image transmission can reach 8 fps when transmitting the compressed 240 × 240 images. The frame rate is actually limited by the data rate of the wireless transmitter. Considering that the knee joint moves quite slowly, this frame rate is acceptable. The refreshing rate of the force data acquisition is 30 Hz.

In the experiment, the spacer (sensing device) is placed in a test platform, which can be used to resemble the situation that the femoral component moves around the spacer during the TKA surgery. As shown in Figure 14b, the test platform includes a base which is used to fix the spacer (sensing device) and a movable femoral component. The femoral component can rotate along a track to resemble the flexion-extension (pitch) rotation around the *O*_C_*X*_C_ axis in the spacer coordinate system. The base to hold the spacer can also rotate to resemble the internal–external (yaw) rotation of the femoral component around the *O*_C_*Z*_C_ axis. The mediolateral distance between the femoral component and the spacer can also be adjusted to resemble the translational displacement. The test platform consists of the rotation and translation tracks with the scales, and the pitch and yaw angles and the translational displacement of the femoral component can be precisely controlled. The sensing devices take the image of the femoral component and record the contact force between the sensing device and the femoral component during the rotation and translation.

### 5.2. Experiment Results of Instant Pose Reconstruction

**Figure 15:**
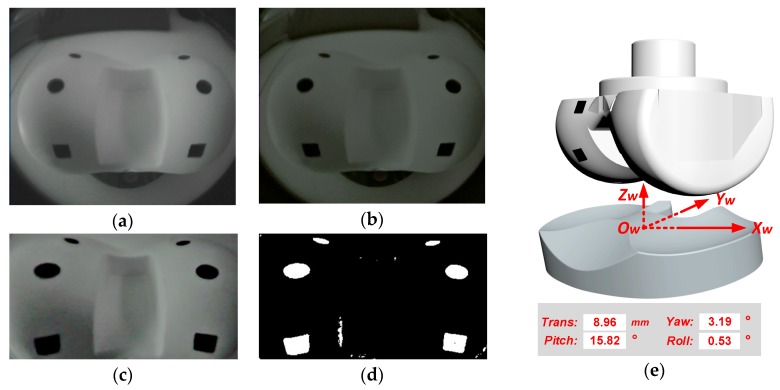
Instant relative pose reconstruction: (**a**) original image, (**b**) after denoising and contrast enhancement, (**c**) after lens distortion correction, (**d**) control point recognition, (**e**) instant pose reconstruction.

The signal processing steps of the instant relative pose reconstruction is shown in Figure 15. Figure 15a gives one typical original image before the processing. Figure 15b–d give the image after the denoising and contrast enhancement, the distortion correction, and the control point recognition, respective. Figure 15e gives the relative pose reconstruction result, including the pitch, yaw, and the roll angles, and the translational distance.

**Figure 16:**
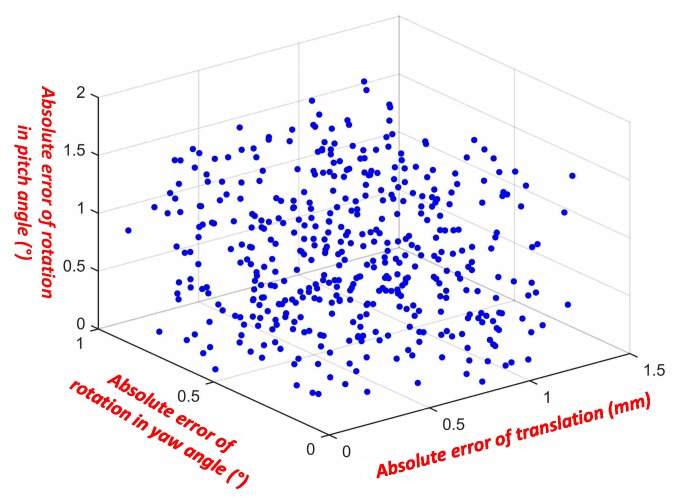
Experimental pose reconstruction errors of flexion-extension (pitch) angle, internal–external (yaw) angle, and mediolateral translation.

To evaluate the pose reconstruction accuracy, more than 400 images of the femoral component are taken by the sensing device, with the flexion-extension (pitch) angle varying from 0° to 90° with a step of 10°, the internal–external (yaw) angle varying from −15° to 15° with a step of 5°, and the mediolateral translation varying from 0 to 30 mm with a step of 5 mm. During the instant pose reconstruction experiment, the femoral component is placed on the test platform. The relative flexion-extension (pitch) angle, internal–external (yaw) angle and translational displacement of the femoral component with respect to the spacer are adjusted, and the motion degrees/distances are read from the scales on the test platform. The measurement errors are obtained by comparing the reconstructed pose to the motion values read from the scales. The detailed pose reconstruction error distribution is shown in Figure 16. With the proposed system, the flexion-extension angle estimation error of 0.01° ± 1.06° (mean ± σ), and the internal–external estimation error is 0.03° ± 0.50° (mean ± σ). As a contrast, in [17], the flexion-extension angle estimation error is 0.0° ± 0.9° (mean ± σ), and the internal–external estimation error is 0.2° ± 1.1° (mean ± σ). The presented vision system has a smaller estimation error. In addition, the most important performance for this system is the absolute maximum error. The absolute maximum flexion-extension (pitch) and internal–external (yaw) reconstruction errors are 1.73° and 1.08°, respectively. And the absolute maximum mediolateral translation reconstruction error is only 1.55 mm. The instant relative pose reconstruction accuracy is summarized in Table 2.

### 5.3. Balance Measurement with Force Sensing

**Figure 17:**
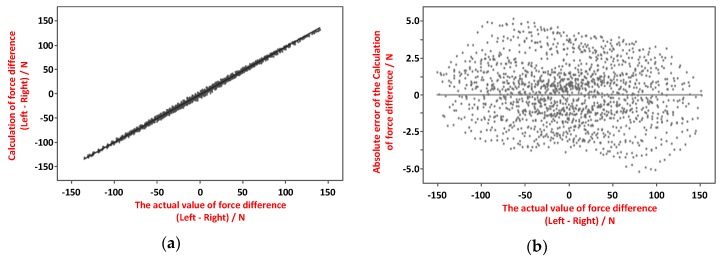
Contact force imbalance measurement: (**a**) measured force difference vs. actual force difference, (**b**) force imbalance measurement error vs. actual force difference.

The force sensing data can be used to determine the contact force between the spacer and the femoral component is balanced between the sides. In the experiment, imbalanced forces are applied to the sides of the spacer (sensing device), and the force sensors in the sensing device measure the force difference (imbalance). Figure 17a shows the measured force imbalance versus the actual force imbalance, and Figure 17b shows the force imbalance measurement error versus the actual force imbalance. The maximum force imbalance measurement error is less than ±5 N, which is adequately small to judge the ligament balance of the knee joint prosthesis.

### 5.4. Kinetic Pose Reconstruction

In this kinetic pose reconstruction experiment, the femoral component is placed in two typical situations, and the relative trajectories of the femoral component “seen” by the spacer are acquired and plotted. Specifically, the femoral component rotates around the *O*_C_*X*_C_ and *O*_C_*Z*_C_ axes of the sensing device slowly with a rotation velocity of ~30° per second, and this rotation velocity is close to that in the TKA procedure in which the surgeons check the trial implants by slowly moving the tibia with respect to the femur.

**Figure 18:**
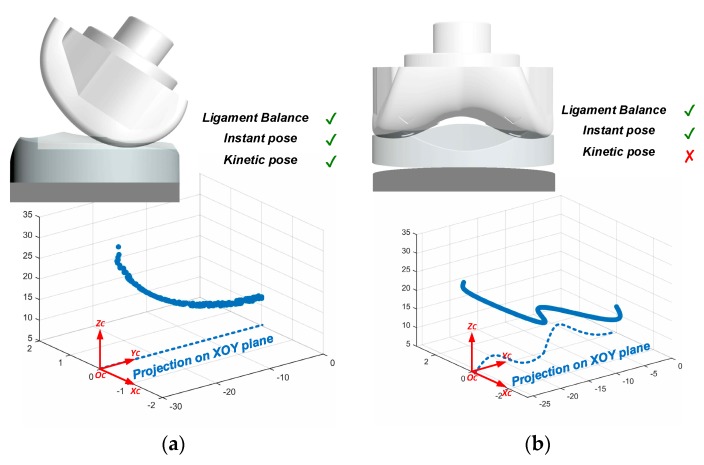
Examples of kinetic pose reconstruction: (**a**) successful surgery gives smooth kinetic trajectory, (**b**) kinetic trajectory finds inappropriate surgery which cannot be found using force measurement or instant pose reconstruction.

As shown in Figure 18a, the femoral component is in an appropriate position. The measured force data shows good ligament balance, the instant relative pose meets the expectation, and the kinetic trajectory is smoot and resides on the *YOZ* plane. 

As shown in Figure 18b, the femoral component is in the good position, but the spacer does not exactly fit the space between the femoral component and tibia component. In this case, both the force measurement and the instant relative pose at some specific point show good result. But the kinetic trajectory shows that relative movement between the spacer and the femoral component has some fluctuation which is does not meet the expectation. This case shows an example of TKA surgery defection that can be found only using the kinetic pose reconstruction.

The experiment results show that it provides more comprehensive evaluation of the TKA surgeries by combining the result of force sensing, the instant pose reconstruction, and the kinetic post reconstruction, rather than that with only the force sensing in [8].

## 6. Conclusions

A wireless visualized measurement system has been proposed and implemented to improve the quality of the TKA surgeries. The system consists of a multimodal sensing device, a wireless data receiver and a multimodal data processing workstation. The multimodal sensing device can take the images of the femoral component of the knee prosthesis and measure the contact force distribution between the spacer and the femoral component. The system is capable of the instant and kinetic prosthesis pose reconstruction based on the image sensing. With the image processing algorithms proposed in this work, the proposed system can provide pose reconstruction with higher accuracy. The absolute reconstruction errors of the flexion-extension rotation angle, the internal–external rotation angle as well as the mediolateral distance between the femoral component and the spacer are less than 1.73°, 1.08° and 1.55 mm, respectively. The force imbalance measurement error is less than ±5N. With the kinetic pose estimation algorithm, the presented system can output a 3D motion trajectory which is not available from other systems that acquire the single modal data such as the force data. The system is used during the surgery as a trial component. The influence on the TKA procedure is quite small. The TKA surgeons can easily use it in the standard clinical procedures.

The current implementation has several limitations that can be improved in the future. The image sensing frame rate is limited to 8 fps due to the limited data rate provided by the transmitter. The wireless transmission distance is limited due to the limited antenna gain. In the future, the transmitter and antenna design will be improved so that the system can provide higher image frame rate and longer wireless communication distance. The device will soon be validated in the real clinical environment. There is the possibility that the data processing algorithm performance may degrade under real situations. The algorithms will be then optimized for the real situations.

## Figures and Tables

**Table 1:** Sensing device performance summary.

Specification		Performance
Typical size (varies with the prosthesis model)		76 mm × 52 mm × 17 mm
Typical weight (varies with the prosthesis model)		25.56 g
Force sensing	Number of sensors	6
	Measurement range (each sensor)	0–45 N
Image sensing	Resolution	240 × 240 or 480 × 480
	Maximum frame rate	8 fps (240 × 240)
Wireless transmitter	Carrier frequency	416 MHz
	Maximum data rate	3 Mbps
Power supply		3 V
Average power consumption		~10 mA@3V
Battery Lifetime(CR2025 Li/MnO2)		>5 h

**Table 2:** Pose Reconstruction Errors.

	Maximum Error (Absolute Value)	Average Error (Absolute Value)	Mean Error	Standard Derivation σ
flexion-extension (pitch)	1.73°	0.67°	−0.01°	1.06°
internal–external (yaw)	1.08°	0.51°	0.03°	0.50°
mediolateral translation	1.55 mm	0.82 mm	−0.04 mm	0.72 mm

## Appendix A

In (17)–(22), the coefficients *a*_i_, *b*_i_, *c*_i_, *d*_i_, *e*_i_, *f*_i_, *g*_i_, *h*_i_, *i*_i_, *j*_i_ (*i* = 1, 2, 3) are listed below:

(A1) $$\{\begin{array}{c} a_{1}=D_{1}^{2}/\left(D_{1}^{2}+D_{2}^{2}\right)\\ b_{1}=\cos^{2}\gamma_{2}-\cos\gamma_{1}\vec{r_{2}}*\vec{r_{3}}\\ c_{1}=a_{1}\sin^{2}\gamma_{2}-\cos\gamma_{1}\cos\gamma_{2}\vec{r_{2}}*\vec{r_{3}}+\cos^{2}\gamma_{2}+\cos^{2}\gamma_{1}-1\\ d_{1}=\vec{r_{2}}*\vec{r_{3}}\\ e_{1}=\cos\gamma_{2}\vec{r_{2}}*\vec{r_{3}}-\cos\gamma_{1} \end{array}$$

(A2) $$n_{1}=a_{1}\sin^{2}\gamma_{2}-\sin^{2}\gamma_{1}$$

(A3) $$\{\begin{array}{c} f_{1}=a_{1}^{2}-a_{1}d_{1}^{2}\\ g_{1}=2\left(a_{1}b_{1}-a_{1}d_{1}e_{1}\right)\\ h_{1}=b_{1}^{2}+2a_{1}c_{1}-a_{1}e_{1}^{2}-n_{1}d_{1}^{2}\\ i_{1}=2\left(b_{1}c_{1}-n_{1}d_{1}e_{1}\right)\\ j_{1}=c_{1}^{2}-n_{1}e_{1}^{2} \end{array}$$

(A4) $$\{\begin{array}{c} a_{2}=D_{1}^{2}/\left(D_{1}^{2}+D_{2}^{2}\right)\\ b_{2}=\cos^{2}\gamma_{2}-\cos\gamma_{3}\vec{r_{4}}*\vec{r_{3}}\\ c_{2}=a_{2}\sin^{2}\gamma_{2}-\cos\gamma_{3}\cos\gamma_{2}\vec{r_{4}}*\vec{r_{3}}+\cos^{2}\gamma_{2}+\cos^{2}\gamma_{3}-1\\ d_{2}=\vec{r_{4}}*\vec{r_{3}}\\ e_{2}=\cos\gamma_{2}\vec{r_{4}}*\vec{r_{3}}-\cos\gamma_{3} \end{array}$$

(A5) $$n_{2}=a_{2}\sin^{2}\gamma_{2}-\sin^{2}\gamma_{3}$$

(A6) $$\{\begin{array}{c} f_{2}=a_{2}^{2}-a_{2}d_{2}^{2}\\ g_{2}=2\left(a_{2}b_{2}-a_{2}d_{2}e_{2}\right)\\ h_{2}=b_{2}^{2}+2a_{2}c_{2}-a_{2}e_{2}^{2}-n_{2}d_{2}^{2}\\ i_{2}=2\left(b_{2}c_{2}-n_{2}d_{2}e_{2}\right)\\ j_{2}=c_{2}^{2}-n_{2}e_{2}^{2} \end{array}$$

(A7) $$\{\begin{array}{c} m=\vec{r_{2}}*\vec{r_{4}}\\ n_{3}=\left(\cos\gamma_{3}\vec{r_{2}}*\vec{r_{4}}-\cos\gamma_{1}\right)\\ p=\left(\cos\gamma_{1}\vec{r_{2}}*\vec{r_{4}}-\cos\gamma_{3}\right)\\ q=\left(\cos\gamma_{1}\cos\gamma_{3}\vec{r_{2}}*\vec{r_{4}}-\cos^{2}\gamma_{1}+\sin^{2}\gamma_{3}\right) \end{array}$$

(A8) $$\{\begin{array}{c} f_{3}=a_{1}a_{2}m\\ g_{3}=m\left(a_{1}b_{2}+a_{2}b_{1}\right)+n_{3}\left(a_{1}d_{2}+a_{2}d_{1}\right)\\ h_{3}=m\left(a_{1}c_{2}+a_{2}c_{1}+b_{1}b_{2}\right)+n_{3}\left(a_{1}e_{2}+b_{1}d_{2}\right)+p\left(a_{2}e_{1}+b_{2}d_{1}\right)+qd_{1}d_{2}\\ i_{3}=m\left(b_{1}c_{2}+b_{2}c_{1}\right)+n_{3}\left(c_{1}d_{2}+b_{1}e_{2}\right)+p\left(c_{2}d_{1}+b_{2}e_{1}\right)+q\left(e_{1}d_{2}+d_{1}e_{2}\right)\\ j_{3}=mc_{1}c_{2}+n_{3}c_{1}e_{2}+pe_{1}c_{2}+qe_{1}e_{2} \end{array}$$
